# Interaction Analysis of a *Plasmodium falciparum* PHISTa-like Protein and PfEMP1 Proteins

**DOI:** 10.3389/fmicb.2020.611190

**Published:** 2020-11-13

**Authors:** Baoling Yang, Xiaofeng Wang, Ning Jiang, Xiaoyu Sang, Ying Feng, Ran Chen, Xinyi Wang, Qijun Chen

**Affiliations:** ^1^Key Laboratory of Livestock Infectious Diseases in Northeast China, Ministry of Education, Key Laboratory of Zoonosis, Shenyang Agricultural University, Shenyang, China; ^2^College of Food Science and Technology, Shenyang Agricultural University, Shenyang, China; ^3^The Research Unit for Pathogenic Mechanisms of Zoonotic Parasites, Chinese Academy of Medical Sciences, Shenyang, China; ^4^CAS Key Laboratory for Biomedical Effects of Nanomaterials and Nanosafety, Institute of High Energy Physics, Chinese Academy of Sciences (CAS), University of Chinese Academy of Sciences, Beijing, China; ^5^College of Basic Sciences, Shenyang Agricultural University, Shenyang, China

**Keywords:** *Plasmodium falciparum*, PHIST, PfEMP1, proteins interactions, molecular dynamic simulation, malaria

## Abstract

*Plasmodium falciparum* extensively remodels host cells by translocating numerous proteins into the cytoplasm of red blood cells (RBCs) after invasion. Among these exported proteins, members of the *Plasmodium* helical interspersed subtelomeric (PHIST) family are crucial for host cell remodeling and host-parasite interactions, and thereby contribute to malaria pathogenesis. Herein, we explored the function of PF3D7_1372300, a member of the PHIST/PHISTa-like subfamily. PF3D7_1372300 was highly transcribed and expressed during the blood stage of *P. falciparum*, and distributed throughout RBCs, but most abundant at the erythrocyte membrane. Specific interaction of PF3D7_1372300 with the cytoplasmic tail of *P. falciparum* erythrocyte membrane protein 1 (PfEMP1) was revealed by immunofluorescence assay, *in vitro* intermolecular interaction assays. The interaction sites of PF3D7_1372300 with PfEMP1 ATS domain were found involved more than 30 amino acids (aa) at several positions. The findings deepen our understanding of host-parasite interactions and malaria pathogenesis.

## Introduction

Malaria is a globally distributed disease that poses a serious threat to public health (Chiodini, 2018). The World Health Organization (WHO) estimated that in 2018, there were 228 million cases of malaria, resulting in 405,000 deaths (WHO, 2019). Among the malaria parasites that infect humans, *Plasmodium falciparum* is the most virulent species (Milner, 2018). *P. falciparum* has a complex life cycle that involves human hosts and female *Anopheles* spp. mosquitoes as vectors (Bryan et al., 2017). Proliferation and differentiation of *P. falciparum* inside erythrocytes take place within ~48 h of infection (Gruring et al., 2011). During intraerythrocytic development, *P. falciparum* exports a large number of proteins into erythrocytes, some of which form a complex network with host proteins, while others are transferred onto the infected red blood cell (iRBC) surface (Maier et al., 2008; Prajapati and Singh, 2013; Oberli et al., 2014; Warncke et al., 2016). At this stage, *P. falciparum* remodels human erythrocytes, which results in structural and physiological alterations and increased rigidity of the erythrocyte membrane. These changes not only help the parasite to evade host immune clearance and support intracellular ion homeostasis and nutrient intake but also cause severe pathogenesis (Mok et al., 2007).

Among the exported proteins, members of the *Plasmodium* helical interspersed subtelomeric (PHIST) protein family are crucial for host cell remodeling (Nilsson et al., 2018). The PHIST gene family was originally targeted using a novel algorithm, ExportPred, which proved superior to other algorithms for the prediction of exported proteins in *Plasmodium* parasites (Sargeant et al., 2006). Genes encoding PHIST proteins are mostly located in subtelomeric regions of most of the chromosomes, and the encoded proteins are distributed in various subcellular locations, including submembrane regions (Warncke et al., 2016). Previous studies identified several PHIST proteins in host-parasite protein interaction complexes (Wahlgren et al., 2017). This family includes 72 protein variants, most of which still have no functional annotations. Based on the number and position of tryptophan residues, *P. falciparum* PHIST protein family members cluster into PHISTa, PHISTb, PHISTc, PHISTb-DnaJ, and PHIST/PHISTa-like subgroups (Sargeant et al., 2006; Claessens et al., 2012). The PHISTa subgroup is unique to *P. falciparum*, while homologous PHISTb and PHISTc sequences are present in other *Plasmodium* lineages, including PHISTb type variants in *Plasmodium vivax* and a single copy of the highly variable PHISTc type variant in rodent malarial parasites (Sargeant et al., 2006). Members share similar structural features, including a classical N-terminal signal sequence followed by PEXEL or VTS motifs, *Plasmodium*-specific signal motifs present in exported proteins (Hiller et al., 2004; Marti et al., 2004). In coordination with the translocon machinery, PHIST proteins are positioned at the appropriate locations (de Koning-Ward et al., 2009). The most characteristic feature of PHIST proteins is the carboxyl terminus PHIST domain, which has a conserved helical structure that mediates most of the interactions with other partner proteins (Warncke et al., 2016). Currently, two PHIST variants, PFE1605w (PHISTb type) and PFI1780w (PHISTc type), have been reported to act as anchors *via* specific interactions with the acidic C-terminal segment (ATS) of *P. falciparum* erythrocyte membrane protein 1 (PfEMP1; Mayer et al., 2012; Oberli et al., 2014). Members of this protein family mediate cytoadherence and severe malaria pathogenesis. PfEMP1 proteins are encoded by the *var* gene, and there are ~60 genes in each *P. falciparum* family (Gardner et al., 2002). Each PfEMP1 molecule is composed of a variable duffy binding-like domain (DBL), a cysteine-rich domain region (CIDR), a transmembrane domain, and a C-terminal ATS (Flick and Chen, 2004; Singh et al., 2006). PfEMP1 proteins are associated with severe malaria pathogenesis because they mediate adhesion between iRBCs and host cells. PfEMP1s are synthesized inside the parasite and are transported to the iRBC surface, but the mechanism of intracellular transportation is not clear (Mayer et al., 2012). However, cytoadhesion of PfEMP1 is directly affected by a protein, which is from the PHISTb subfamily, and deletion of PFE1605w severely diminishes iRBC adhesion to the CD36 receptor, although expression of PfEMP1 is not affected (Oberli et al., 2016). This indicates that PFE1605w type PHIST is critical for the structure stability of PfEMP1 on iRBCs. However, the structure and function of PHIST/PHISTa-like subgroups remain poorly understood.

In this study, we systematically characterized a PHIST/PHISTa-like protein (PF3D7_1372300), which was expressed throughout the erythrocytic stage in iRBCs. The protein has a typical four-helix bundle structure, it interacts with PfEMP1 (PF3D7_0800200), and interaction sites were predicted using molecular dynamics (MD) simulation. The findings greatly expand our knowledge of this important protein family in the context of host-parasite interactions.

## Materials and Methods

### Ethical Statement

All procedures performed on animals (rats and rabbits) were conducted according to the animal husbandry guidelines of Shenyang Agricultural University. Protocols for experiments were reviewed and approved by the Experimental Animal Committee and the Ethical Committee of Shenyang Agricultural University, Shenyang, China.

### Parasite Culture

The *P. falciparum* 3D7 strain was cultured in human O^+^ erythrocytes with 5% serum and 0.25% Albumax II, according to the standard methods reported previously (Florens et al., 2004; Hiller et al., 2004). Briefly, parasites were synchronized by three rounds of treatment at 4 h post-invasion with 5% sorbitol and harvested at 8, 16, 24, 32, 40, and 48 h post-infection.

### Expression of Recombinant Proteins and Generation of Specific Antibodies

DNA encoding PF3D7_1372300 (residues 27-206) and PF3D7_0800200 (residues 2,466-2,858) were PCR-amplified from *P. falciparum* cDNA using pairwise primers listed in Supplementary Table S1. The PF3D7_1372300 and PF3D7_0800200 PCR products were cloned into the pET-28a vector, while the PF3D7_1372300 PCR product was cloned into the pGEX-4T-1 vector. Constructs were expressed in *Escherichia coli* BL21 (DE3) cells as His-tagged and GST-tagged recombinant proteins and were purified according to the manufacturer’s instructions (Ramaprasad et al., 2012). SDS-PAGE and Western blotting were used to evaluate the purified recombinant proteins. Two female New Zealand white rabbits and four female Sprague Dawley rats were immunized with His-tagged recombinant proteins emulsified with Freund’s adjuvant. Immunizations were performed four times every 2 weeks, and antisera were collected 10 days after the final immunization. IgGs were purified from the immune sera using Protein G Sepharose 4 Fast Flow Resin (GE Healthcare) and Protein A Sepharose 4 Fast Flow resin (GE Healthcare), respectively. The total proteins of iRBCs and RBCs were used to detect the specificity of the antibodies by Western blotting.

### RT-qPCR Transcriptional Analysis of the PF3D7_1372300 Gene

Total RNA from highly synchronized parasites at six post-infection time points was extracted using TRIzol reagent (Invitrogen, United States) according to the manufacturer’s instructions. DNase I (TaKaRa, China) was used to remove genomic DNA from RNA samples, and next, oligo (dT) primer and reverse transcriptase were immediately employed. RT-qPCR was carried out using cDNA templates and specific primers (Supplementary Table S2) on a QuantStudio 6 Flex Real-Time PCR System (Applied Biosystems, United States) with SYBR Premix Ex Taq (TaKaRa). The seryl-tRNA synthetase gene (PF3D7_1205100; LaCount et al., 2005) served as an internal control for normalization because it is stably expressed during the blood stage of the parasite. Data are expressed as means ± SD of at least three independent experiments.

### Expression and Localization Analysis of the Native Protein in Parasites by Western Blotting and Indirect Immunofluorescence Assay

Infected red blood cells at six time-points post-infection were collected and dissolved in SDS-PAGE loading buffer after ultrasonic disruption, and the proteins were separated by 10% SDS-PAGE and were stained by Coomassie Brilliant Blue. Meanwhile, proteins were transferred to a 0.2 μM polyvinylidene fluoride membrane (Bio-Rad, United States). Membranes were blocked with 5% skim milk (BD, United States) for 2 h at 37°C followed by incubation with rabbit anti-PF3D7_1372300 IgG (1:1,000 dilution) for 12 h at 4°C. Rabbit anti-Hsp70 IgG (1:1,000 dilution) was used for normalization. Membranes were further incubated with horseradish peroxidise (HRP)-conjugated goat anti-rabbit IgG (H + L; 1:10,000 dilution; Zsbio, China) for 1 h at 37°C and were visualized with ECL Western blotting (Solarbio, China) using an Azure Biosystems C300 Image Analyzer (Azure, United States).

Thin blood smears of iRBCs at different stages were fixed with cold methanol in −80°C for 15 min and were blocked in 5% skim milk (BD) for 1 h at 37°C. Smears were then incubated with rabbit anti-PF3D7_1372300 antibody (1:100 dilution) at 4°C for 12 h, followed by incubation with Alexa Fluor 488-conjugated goat anti-rabbit IgG at a 1:600 dilution (Invitrogen) at 37°C for 1 h. Parasite nuclei were stained with ProLong Gold Antifade Mountant and DAPI (Invitrogen) at room temperature for 5 min in the dark. High-resolution images were captured with a confocal laser scanning microscope (Leica, SP8, Germany).

### Kinetic Analysis of the Interaction Between PF3D7_1372300 and PfEMP1 ATS

An Octet K2 instrument was used to probe the kinetics of PF3D7_1372300-GST and PF3D7_0800200-His. GST and ATS were served as a negative control. ATS-His was loaded onto Ni-NTA biosensors, and PF3D7_1372300-GST at different concentrations were prepared in phosphate-buffered saline (PBS) containing 0.02% Tween-20.

Kinetic studies were performed to determine the binding affinities of PF3D7_1372300-GST and GST (diluted from 1,000 to 62.5 nM) with ATS-His. Association and dissociation were measured for 5 min, and kinetic parameters and affinities were calculated from a non-linear global fit of the data using Octet Data Analysis software version 7.0 (ForteBio, United States).

### Dot Blot Assays

Purified ATS bait protein was immobilized on an nitrocellulose (NC) membrane, total protein from iRBCs containing the PF3D7_1372300 target protein (prey) and total protein from RBCs (negative control) were added and incubated for 1 h, respectively. The NC membrane was blocked with 3% BSA in PBS for 1 h, followed by incubation with rat anti-PF3D7_1372300 IgG primary antibodies, and then with HRP-conjugated anti-rat secondary antibodies for 1 h. Meanwhile, purified PF3D7_1372300 bait protein was immobilized on an NC membrane, and total protein from iRBCs containing the ATS target protein (prey) and total protein from RBCs (negative control) were added. The incubation and blocking conditions are as shown above, followed by incubation with rat anti-ATS IgG primary antibodies, and then with HRP-conjugated anti-rat secondary antibodies for 1 h. Dot intensities were measured using ImageJ software for the analysis of binding by plotting on a graph.

### Co-localization of PF3D7_1372300 and ATS

The rabbit IgG of PF3D7_1372300 (1:500) and the rat IgG of ATS (1:1,000) were used to detect the co-localization of the two proteins by indirect immunofluorescence. Anti-ATS rat IgG is stored in our laboratory. The specific method was as mentioned above.

### Molecular Dynamics Simulation

The 3D structure of PF3D7_1372300 was predicted by Itasser and Modeller softwares. The structure of PfEMP1 was generated by homology modeling. The structure of the protein complex was obtained *via* docking, and the five most promising poses were selected. Finally, MD simulations were carried out for each complex structure.

### Verification of Interaction Sites Between PF3D7_1372300 and the ATS Region of PfEMP1 (PF3D7_0800200)

Recombinant proteins PF3D7_1372300-(27-129)-GST and PF3D7_1372300-(130-206)-GST were purified according to the methods described above. SDS-PAGE and Western blotting were used to evaluate the purified recombinant proteins, and kinetic studies were performed to determine the binding affinities between ATS-His and the two GST-tagged recombinant proteins.

## Results

### Sequence Characteristics of the PF3D7_1372300 Protein

**Figure 1:**
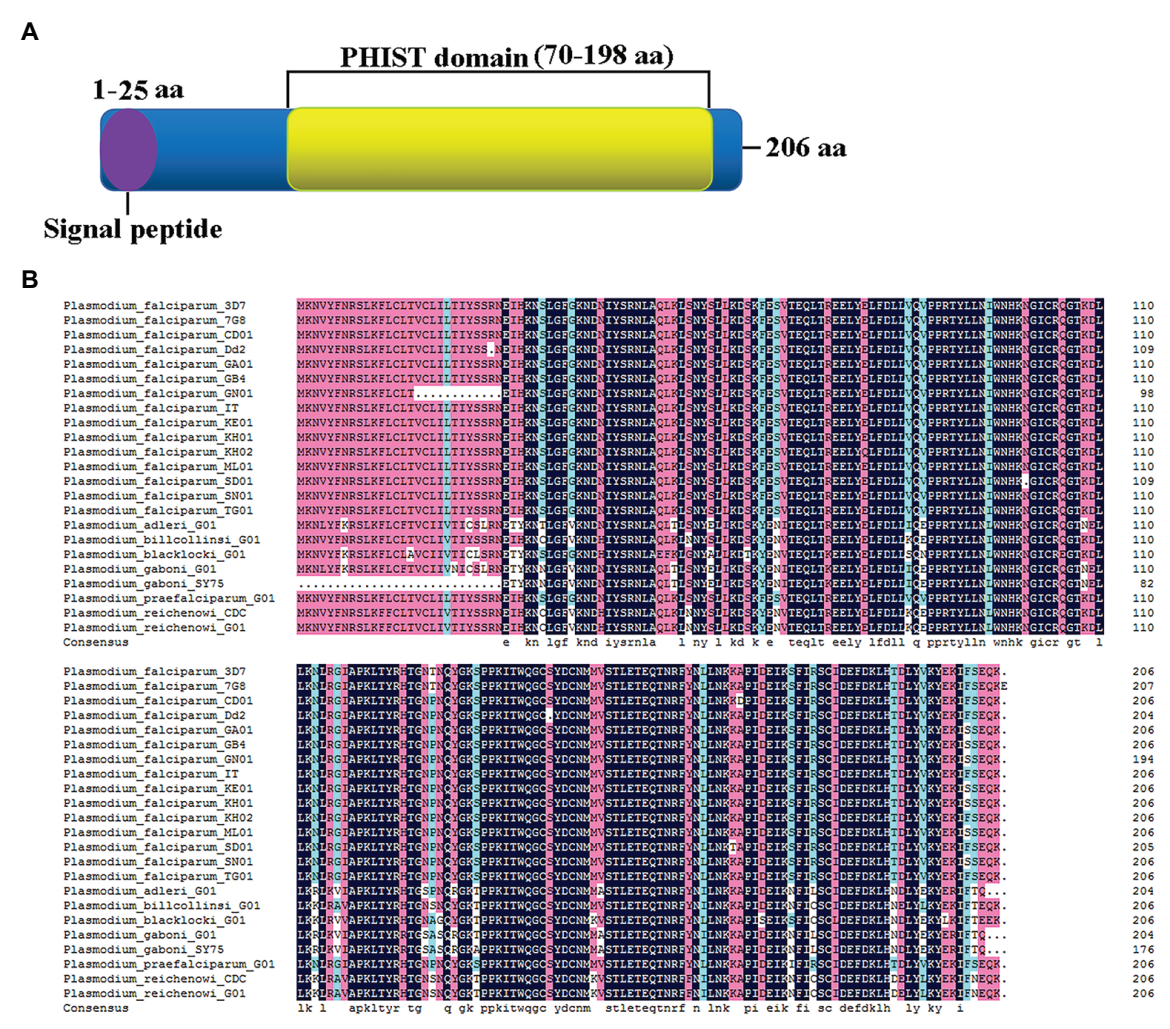
Sequence characteristics of PF3D7_1372300. **(A)** Schematic structure showing the signal peptide (purple) at the N-terminus and a large *Plasmodium* helical interspersed subtelomeric (PHIST) domain (yellow) from the middle to the C-terminus. **(B)** Alignment of PF3D7_1372300 and homologs from different *Plasmodium falciparum* strains and *Plasmodium* spp. that infects gorillas or chimpanzees.

The amino acid sequence of the PF3D7_1372300 protein includes a PHIST domain in the C-terminal region and a 25 amino acid signal peptide domain at the N-terminus, indicating that the protein is secreted from parasites (Figure 1A). Sequence alignment with homologs from other species revealed ~93% sequence identity with different *P. falciparum* strains and *Plasmodium* spp. that infects gorillas or chimpanzees, including *Plasmodium adleri*, *Plasmodium billcollinsi*, *Plasmodium blacklocki*, *Plasmodium gaboni*, *Plasmodium praefalciparum*, and *Plasmodium reichenowi* (Figure 1B).

### Purification of Recombinant Proteins and Detection of Antibodies Specificity

**Figure 2:**
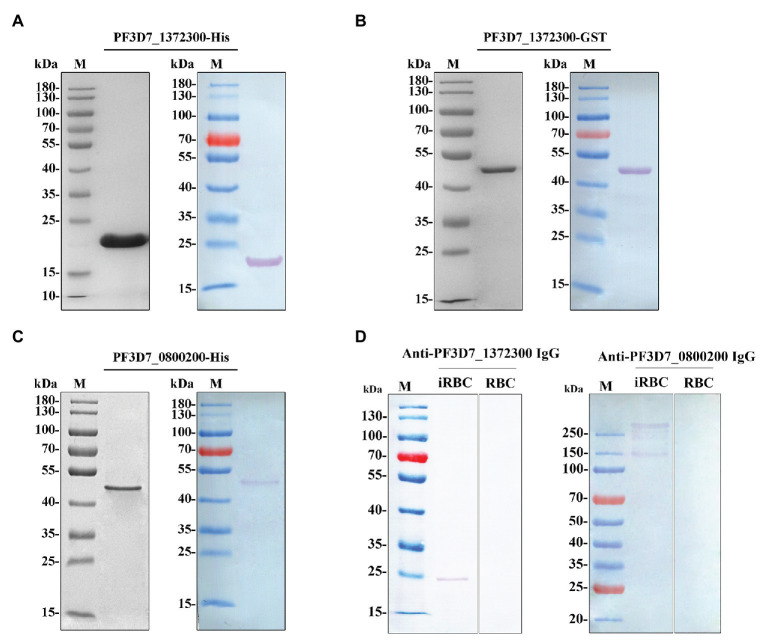
**(A–C)** Purified PF3D7_1372300 (27-206 aa)-His, PF3D7_1372300 (27-206 aa)-GST, and PF3D7_0800200 [acidic C-terminal segment (ATS) domain, 2,466–2,858 aa]-His proteins analyzed by 10% SDS-PAGE (left) and Western blotting (right). A single protein band was observed with a molecular mass of 21, 46, and 47 kDa. **(D)** Western blotting analysis of the antibody specificity of PF3D7_1372300 and PF3D7_0800200 with total protein of infected red blood cells (iRBCs) and red blood cells (RBCs), respectively.

Recombinant proteins of both PF3D7_1372300 and the ATS of PfEMP1 (PF3D7_0800200) were generated (Figures 2A–C). Rabbit anti-PF3D7_1372300 IgG and rat anti-ATS IgG specifically detected the native PF3D7_1372300 protein and all PfEMP1 variants from the total proteins of iRBCs, respectively, and both the two antibodies could not detect proteins from the RBCs (negative control; Figure 2D).

### The PF3D7_1372300 Gene Is Transcribed and Expressed Throughout the Blood Stage of *P. falciparum*

**Figure 3:**
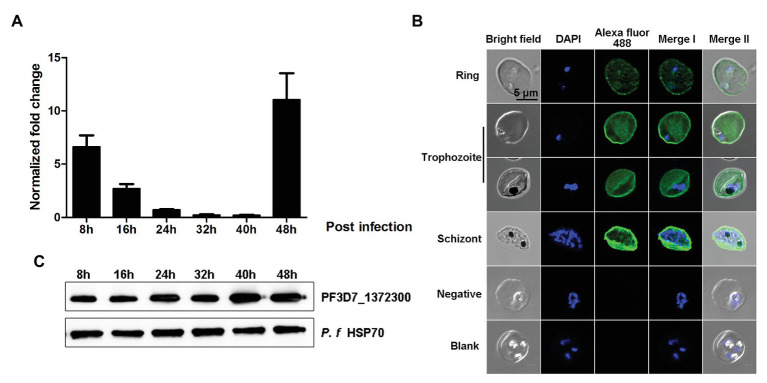
Gene transcription and spatial distribution of the PF3D7_1372300 protein. **(A)** Quantitative real-time PCR was used to measure PF3D7_1372300 gene transcription by the 2^−ΔCt^ method, and the seryl-tRNA synthetase gene (PF3D7_1205100) served as an internal control for normalization. Results are mean ± SD of three independent experiments. **(B)** Indirect immunofluorescence of PF3D7_1372300. Rabbit anti-PF3D7_1372300 antibody served as the primary antibody, IgG from healthy rabbits was used as a negative control, and buffer without antibody served as a blank control. Parasite nuclei were stained with DAPI. **(C)** Western blotting analysis of the expression of native PF3D7_1372300 during the whole blood stage. Hsp70 protein was used as an internal reference to ensure consistent loading, and rabbit anti-PF3D7_1372300 IgG served as the primary antibody. Scale bar = 5 μm.

To investigate transcription and expression of the PF3D7_1372300 gene in the *P. falciparum* 3D7 strain during the blood stage, highly synchronized parasites at six time points (8, 16, 24, 32, 40, and 48 h post-invasion) were collected for gene transcription analysis by quantitative real-time PCR (qPCR). The results showed that the PF3D7_1372300 gene was transcribed throughout the blood stage, reached a peak at ~48 h post-invasion, moreover, and transcription remained high during the ring stage (Figure 3A).

Expression of the PF3D7_1372300 protein was also confirmed by both immunofluorescence assay (IFA) and Western blotting. The IFA results revealed dotted fluorescence on the membrane of iRBCs but also in the cytoplasm, during the early stage of infection. As parasites developed, expression of the protein became more extensive. The fluorescence was distributed throughout the iRBCs, and the fluorescence which was located close to the iRBCs membrane was stronger than that in the cytoplasm of the iRBCs (Figure 3B). The results of Western blotting were consistent with the RT-PCR and IFA data (Figure 3C).

**Figure 4:**
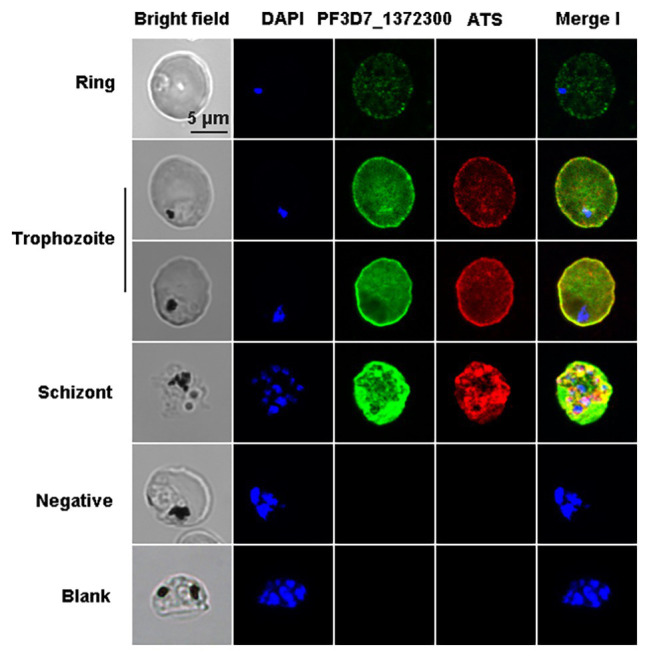
Co-localization of PF3D7_1372300 and ATS using indirect immunofluorescence. Smears of methanol-fixed *Pf*3D7-infected erythrocytes were incubated with anti-PF3D7_1372300 rabbit IgG (1:500) and anti-ATS rat IgG (1:1,000), followed by incubation with Alexa Fluor-conjugated secondary antibody (Alexa Fluor 488, rabbit; Alexa Fluor 594, rat). IgG from healthy rabbits and rats were used as negative controls, and buffer without antibody served as a blank control. Parasite nuclei were stained with DAPI.

The co-localization results showed that the two proteins have fluorescence overlap at the erythrocyte membrane during the trophozoite and schizont stages (Figure 4).

### Binding Between PF3D7_1372300 and the PfEMP1 ATS

**Figure 5:**
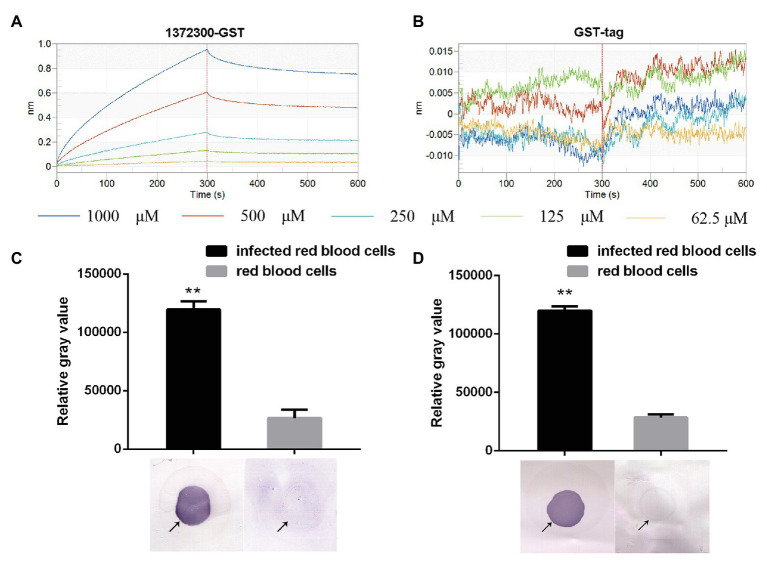
Affinity between PF3D7_1372300 and ATS measured using a ForteBio system. **(A)** PF3D7_1372300-GST. **(B)** GST-tag. Curves correspond to the association and dissociation of PF3D7_1372300-GST/GST-tag at various concentrations and ATS anchored to the sensor chip. The curves can be used to determine KD, Kon, and Kdis. **(C,D)** Affinity between PF3D7_1372300 and ATS measured by dot blot. The arrow shows the position of the droplet. The experiment was repeated in triplicate, and the average relative gray value was calculated by ImageJ software. Statistical analysis was performed using ANOVA. Differences were considered statistically significant at *p* ≤ 0.05 (compared with the control red blood cell group, ^**^p ≤ 0.01).

Kinetic studies were performed to determine the binding affinities between PF3D7_1372300 and ATS. PF3D7_1372300 fused to glutathione-S-transferase (PF3D7_1372300-GST) and GST alone was diluted from 1,000 to 62.5 nM to capture ATS loaded onto the sensors (Figures 5A,B). The Octet K2 system monitored the binding reaction between pairs of proteins in real time, and the binding rates (kon) and dissociation rates (kdis) are listed in Supplementary Table S3. To understand the binding affinity value, the equilibrium dissociation constant (KD) was calculated as Kdis/Kon. A KD value between 1.0E-03 and 1.0E-12 M indicates interaction between two molecules. From the results (Supplementary Table S3), the KD value for binding of PF3D7_1372300-GST to ATS was 1.19E-07, and the KD value for GST binding to ATS (negative control) was <1.0E-12. This indicates a strong affinity between PF3D7_1372300 and ATS.

Binding of the two proteins was further investigated using dot blot assays. From the results, we can conclude that PF3D7_1372300 in the total protein sample from iRBCs successfully captured PfEMP1 ATS on the blot membrane, while negative control failed to capture PfEMP1 ATS (Figure 5C). At the same time, the PfEMP1 ATS in the total protein sample from iRBCs successfully captured PF3D7_1372300 on the blot membrane, while negative control failed to capture PF3D7_1372300 (Figure 5D).

### Molecular Dynamics Simulation

**Figure 6:**
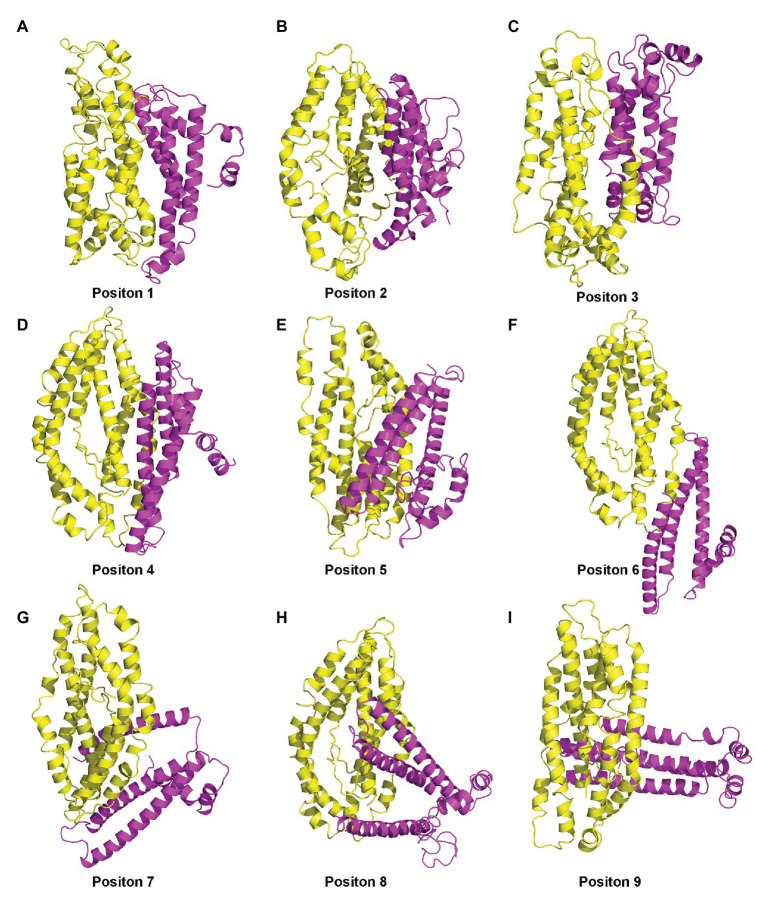
**(A–I)** Nine complex structures screened out by molecular docking.

The sequence from residues 27 to 206 of PF3D7_1372300 was used to predict the 3D structure of PF3D7_1372300, and three structures were obtained by Itasser and Modeller softwares (Supplementary Figures S1A **–** C; Webb and Sali, 2016). PfEMP1 is a large transmembrane protein of 2,858 amino acids (aa) residues, but we only considered the ATS region on the inner part of the membrane. The PfEMP1 sequence from residues 2,486 to 2,759 was used to build the 3D structure using Modeller. Two templates (5MI0 and 2LKL) were identified with sequence identity of 18% and 69%, respectively. The final model of the ATS structure was obtained by further refinement using energy minimization (Supplementary Figure S1D). The two proteins were then docked together using the Zdock server (Pierce et al., 2014), and version 3.0.2 was selected, without predefining the interacting and blocking residues. Among the top 100 complex structures, the top nine were initially screened, and representative structures of the five most abundant clusters (Positions 1–5) were selected as the initial structure for MD simulations (Figure 6).

All MD simulations were performed using the NAMD 2.10 software package (Phillips et al., 2005) and the CHARMM36m protein force field (Huang et al., 2017). The structures were solvated with water molecules (TIP3P model) and 0.15 mM NaCl, and then subjected to energy minimization. For all simulations, a time step of 2 fs was used, along with periodic boundary conditions and Particle-Mesh-Ewald (PME) for electrostatics. The systems were heated to 300 K with constraining of Cα atoms only for 1 ns. The systems were equilibrated for another 1 ns without constraints before analysis. Finally, each system was run for 25, 35, and 50 ns. Parameters for all simulations were temperature 300 K, switching distance 10 Å, switching cutoff 12 Å, pairlist distance 14 Å, Langevin damping coefficient 1 ps^−1^, and Langevin pressure control with a target pressure of 1.01325 bar.

**Figure 7:**
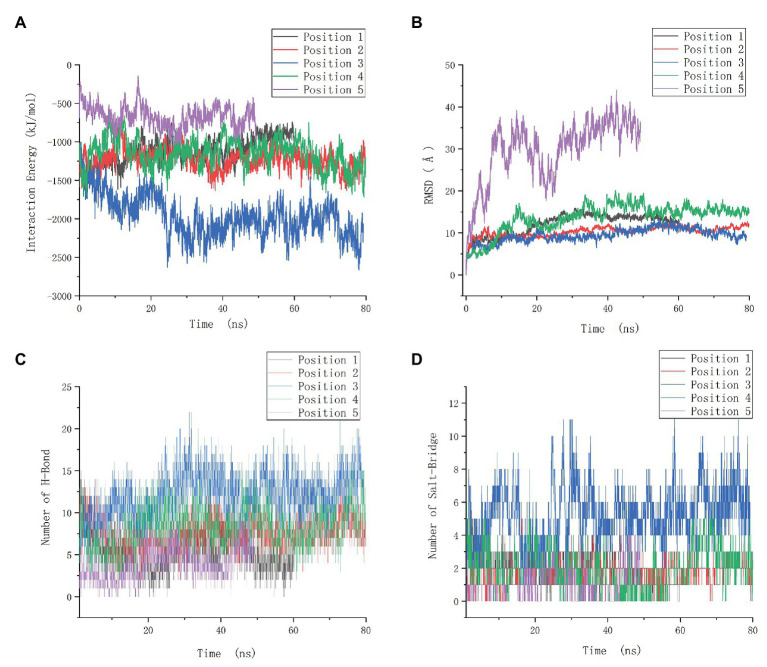
Molecular dynamics data of the positions 1–5. **(A)** Interaction energy of five positions. **(B)** Root mean square deviation (RMSD) of five positions. **(C)** The number of H-bonds of five positions. **(D)** The number of salt-bridges of five positions.

**Figure 8:**
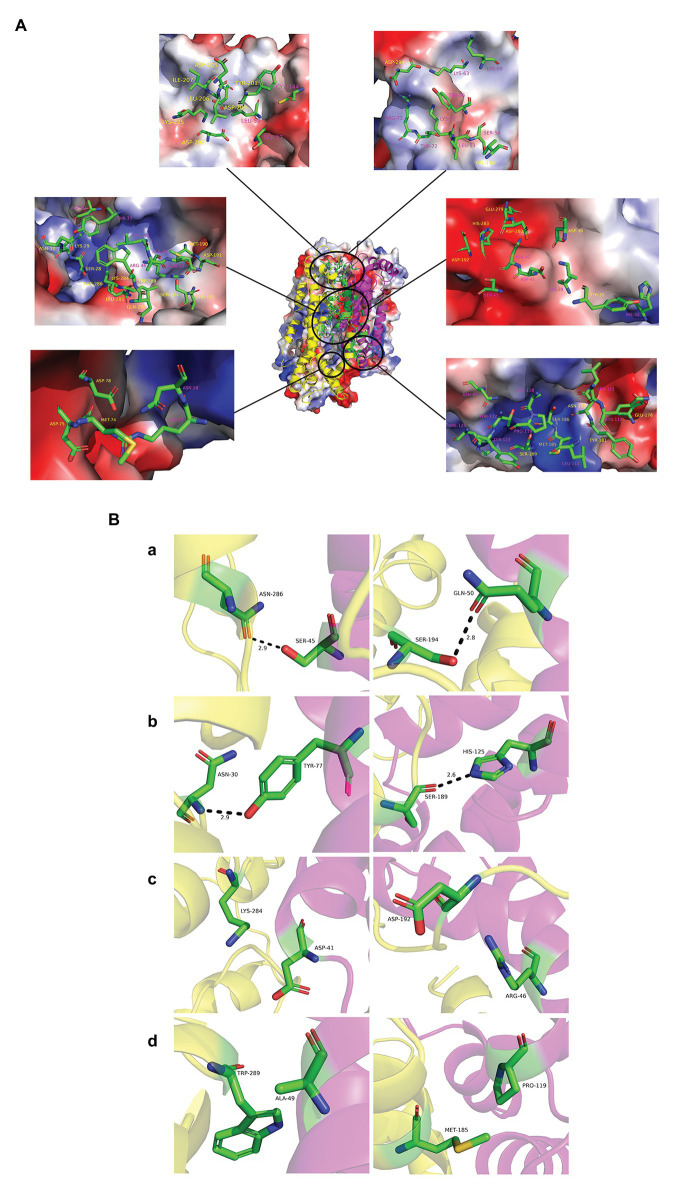
Interaction modes of PF3D7_1372300 and ATS. **(A)** Structure of position 3 and its electrostatic surface were shown in the center. Circles are the sites involved in interactions between the two proteins. The close-up view shows PF3D7_1372300 (purple) and ATS (yellow) residues involved in interactions in electrostatic surface representation. All amino acids are labeled in the figure and shown in stick representation. Carbon atoms are colored green, nitrogen atoms are blue, and oxygen atoms are red. **(B)** PF3D7_1372300 (purple) and ATS (yellow) residues involved in interactions. Rows **(a,b)** show the amino acids involved in the formation of hydrogen bonds (shown as black dotted lines). Row **(c)** shows the amino acids involved in salt bridge formation. Row **(d)** shows the amino acids involved in hydrophobic interactions.

The interaction energy of the two proteins in each system was calculated as the sum of electrostatic and Van der Waals energy and was averaged for the whole trajectories. And the root mean square deviation (RMSD) was further calculated, which is an important indicator for measuring the stability of the system. Position 3 yielded the strongest interaction energy (−1,965.03 kJ/mol) and the lowest RMSD (9.4 Å; Figures 7A,B; Supplementary Table S4). And the preliminary results showed that position 3 forms the most chemical bonds (Figures 7C,D; Supplementary Table S4). Thus, position 3 was retained for subsequent analyses. We further identified H-bonds, salt-bridges, and hydrophobic contacts between PF3D7_1372300 and ATS in the position 3 analysis. The geometrical criteria of H-bonds were set as distance cutoff 3.5 Å and angle cutoff 30. The threshold of salt bridges was a distance between the carboxyl group (COO^−^) and the amino group (NH^3+^) less than 4.0 Å. Hydrophobic contacts between proteins were defined as a distance between heavy atoms in the side chains of residues Ala, Val, Leu, Ile, Met, Phe, Pro, and Trp less than 4.0 Å, and all the sites involved interactions (Figure 8A). Some of the amino acids involved in H-bonds, salt bridges, and hydrophobic interactions are shown in detail in Figure 8B.

### Verification of Interaction Sites

**Figure 9:**
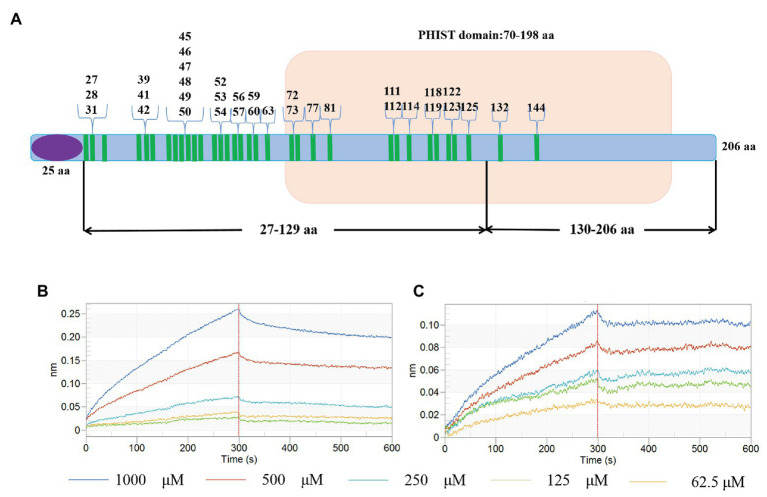
Schematic diagram of interaction sites and interaction verification. **(A)** The blue bar represents PF3D7_1372300, the purple ellipse represents the signal peptide, the part with the light brown background is the PHIST domain, and the green bar represents the amino acids involved in interactions. **(B,C)** Binding affinity for PF3D7_1372300-(27-129)-GST with ATS **(B)** and PF3D7_1372300-(130-206)-GST with ATS **(C)** measured using a ForteBio system.

In order to verify the interaction sites, we divided the PHIST domain of PF3D7_1372300 into two fragments as shown in Figure 9A, and expressed and purified the two corresponding recombinant proteins (Supplementary Figures S2A,B). Kinetic analysis results for the variants are shown in Figures 9B,C and Supplementary Table S5. The KD value between PfEMP1 ATS and PF3D7_1372300-(27-129)-GST was 1.273E-07 was compared with 2.261E-08 between PfEMP1 ATS and PF3D7_1372300-(130-206)-GST. Thus, both PF3D7_1372300-(27-129)-GST and PF3D7_1372300-(130-206)-GST were interacted with PfEMP1 ATS, but the affinity between ATS and PF3D7_1372300-(27-129)-GST was stronger. This result is consistent with MD simulation data. As shown in Figure 9A, PF3D7_1372300-(27-129)-GST contains more sites that interact with PfEMP1 ATS.

## Discussion

For the malaria parasite *P. falciparum* to thrive inside human erythrocytes, it must modulate host cells, and this involves networks of protein-protein interactions. A novel exported PHIST protein was recently linked to parasite biology. The functional diversity of PHIST proteins is highlighted by their involvement in PfEMP1 expression, trafficking, and switching, as well as cytoadherence, gametocytogenesis, host cell modification, and generation of extracellular vesicles (EVs; Haldar and Mohandas, 2007; Maier et al., 2009). Several members of the PHIST family are reportedly essential for parasite survival, which highlights their significance in malaria biology (Maier et al., 2008; Zhang et al., 2018).

Different PHIST members are located in distinct subcellular regions. Several are exported to the iRBC surface (Oberli et al., 2014; Tarr et al., 2014; Kumar et al., 2018), while PFE1605w comigrates with PfEMP1 within iRBCs before being located at knobs, where it likely functions as an anchor of PfEMP1 (Oberli et al., 2014). However, PFI1780w was found to be uniformly distributed along the iRBC membrane, and completely absent from knobs (Oberli et al., 2014), while PF3D7_0424000 and PF3D7_0731100 are located to Maurer’s clefts (MCs; Maier et al., 2008; Kumar et al., 2018). Although MCs appear to be the final destination of PF3D7_0424000, PF3D7_0731100 was also observed in iRBC-derived EVs (Regev-Rudzki et al., 2013) that carry several other PHIST proteins (Abdi et al., 2017). The presence of PHIST proteins at various locations in host cells indicates that they are potential interaction partners for specific proteins.

In the present study, we studied PF3D7_1372300, a PHIST/PHISTa-like protein with an unknown function. Sequence alignment revealed high similarity not only with homologs in *P. falciparum* strains but also in *Plasmodium* spp. that infects gorillas or chimpanzees. This indicates that the protein emerged after evolving in different *Plasmodium* parasite species, and it is different from PHISTa subgroup, which is unique to *P. falciparum* (Figure 1). The PF3D7_1372300 gene was highly transcribed throughout the blood stage of *P. falciparum* (Figure 3A), consistent with a previous genome-wide transcription study (Bozdech et al., 2003). Western blotting results showed that the protein was also expressed throughout the blood stage (Figure 3C). Comparison of expression at the gene and protein level indicated inconsistencies, which may indicate that expression of PHIST proteins was delayed after transcription (Foth et al., 2011).

Immunofluorescence assay experiments with a PF3D7_1372300 protein-specific antibody revealed dotted fluorescence on the iRBCs during the early stages of infection, and the fluorescence intensity was stronger in the trophozoite and late stages, where fluorescence was mainly located on the erythrocyte membrane (Figure 3B). Co-localization of PF3D7_1372300 and ATS revealed that PF3D7_1372300 reached the rim of the iRBC membrane before PfEMP1. In the early stage, the dotted fluorescence signals of PHIST protein were scattered distributed throughout the iRBCs; however, the ATS protein showed no fluorescence signal. During the trophozoite and late stages, significant overlap of fluorescence signals was observed on the erythrocyte membrane (Figure 4), unlike previous reports of PFE1605w co-translocated with a PfEMP1 protein (Oberli et al., 2014). We cannot rule out the possibility of variant-specific interactions between different PHISTs and PfEMP1s, especially given the large number of PfEMP1 and PHIST families.

To probe interactions between the two proteins, a ForteBio Octet K2 biomolecular interaction analysis system was applied that uses biofilm interference technology. This approach measures corresponding changes in the interference pattern of the intensity of the interference wave and the wavelength of emitted light to provide binding efficiency or concentration information for biomolecules interacting in real time. The technique can probe intermolecular interactions in a rapid and high-throughput manner. Our protein interaction and dot blot results confirmed that PF3D7_1372300 interacts strongly with the ATS region of PfEMP1 (Figure 5).

In the present study, interaction sites on PF3D7_1372300 that bind the ATS of PfEMP1 were explored using MD simulation. A total of 34 PF3D7_1372300 aa residues were predicted to potentially participate in these interactions (Figure 8), and these interaction sites were preliminarily verified (Figure 9). Furthermore, 36 aa residues of PfEMP1 ATS were predicted to participate in these interactions (Supplementary Figure S3). The residues enclosed by a yellow box correspond to conserved interaction sites of ATS predicted in previous studies (Mayer et al., 2012), and five of the residues identified in our MD simulations are located in this predicted conserved interaction region.

In summary, we explored the transcription and expression of a novel PHIST protein (PF3D7_1372300) of *P. falciparum*. We verified the interaction between PF3D7_1372300 and PfEMP1 ATS, and further identified the interaction sites. The findings greatly expand our knowledge of the PHIST protein family in host-parasite interactions, and provide a basis for exploring the functions of this important protein family.

## Data Availability Statement

The original contributions presented in the study are included in the article/Supplementary Material, further inquiries can be directed to the corresponding author.

## Ethics Statement

The animal study was reviewed and approved by the Experimental Animal Committee and the Ethical Committee of Shenyang Agricultural University, Shenyang, China.

## Author Contributions

BY performed most of the experiments, analyzed the data, and wrote the first draft of the manuscript. XiaW and XinW performed the modeling of protein-protein interaction. NJ mentored the study. XS, YF, and RC performed the IFA analysis. QC conceived the study, analyzed that data, and finalized the manuscript. All authors contributed to the article and approved the submitted version.

### Conflict of Interest

The authors declare that the research was conducted in the absence of any commercial or financial relationships that could be construed as a potential conflict of interest.

## Abbreviations

ATS: Acidic C-terminal segment
EVs: Extracellular vesicles
IFA: Immunofluorescence assay
iRBCs: Infected red blood cells
MCs: Maurer’s clefts
PfEMP1: *Plasmodium falciparum* erythrocyte membrane protein 1
PHIST: *Plasmodium* helical interspersed subtelomeric
PME: Particle-Mesh-Ewald
qPCR: Quantitative real-time PCR
RMSD: Root mean square deviation
