# Supplementary material for: Interaction Analysis of a Plasmodium falciparum PHISTa-like Protein and PfEMP1 Proteins
Source: Front Microbiol. 2020 Nov 13;11:611190. doi: 10.3389/fmicb.2020.611190 (PMC7691434; doi:10.3389/fmicb.2020.611190)
Supplement: Supplementary file 1 [file Data_Sheet_1.pdf]

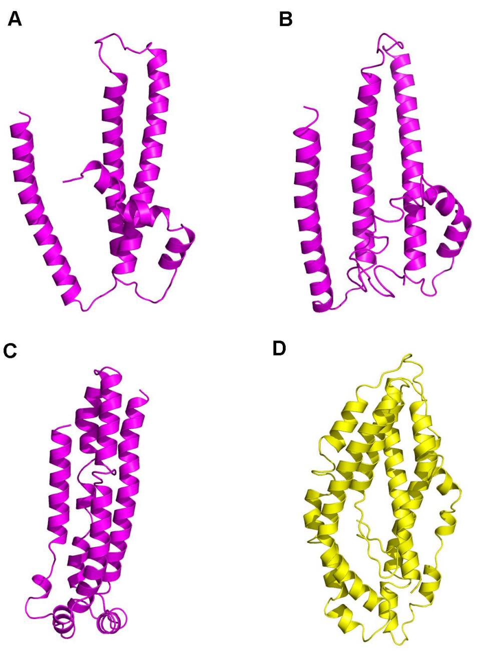

Figure S1. (A) The 3D structures of PF3D7_1372300 obtained by Itasser software. (B) The relaxed structure of PF3D7_1372300 from Itasser's prediction. (C–D) The structures of PF3D7_1372300 and ATS built using Modeller, respectively.

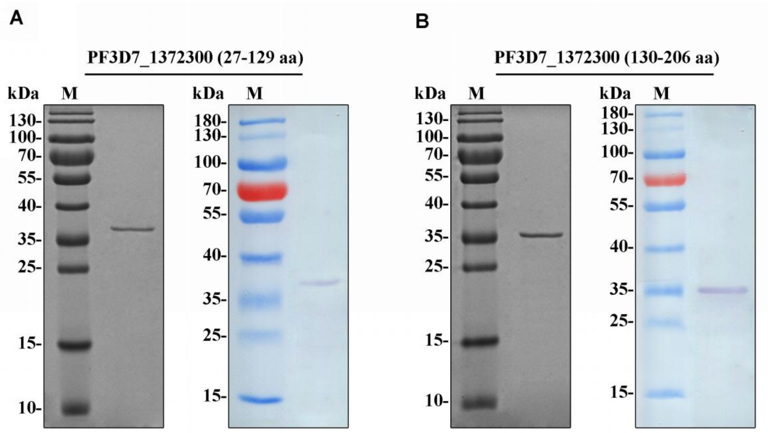

Figure S2. (A – B) Analysis of purified PF3D7_1372300 (residues 27 – 129) and PF3D7_1372300 (residues 130 – 206) by SDS-PAGE (left) and western blotting (right).

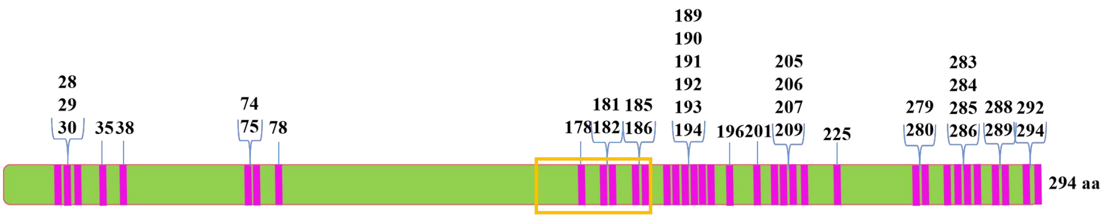

Figure S3. Schematic diagram of interaction sites on ATS. The green bar represents the ATS protein, the deep pink bar represents the amino acids involved in interactions, and the yellow box shows the predicted conserved interaction sites of ATS in previous studies. Five of the residues identified in our MD simulations are located in this predicted conserved interaction region.

Table S1. The primer sequences for cloning the gene fragments encoding the recombinant proteins expressed in *E. coli*.

Gene name	Primers
PF3D7_1372300-His	F:CATGCCATGGGACGAAATGAGATACATAAA<br>R:CCGCTCGAGTTTTGTTCACTAAATATTTTTTCA
PF3D7_1372300-GST	F:CGCGGATCCCGAAATGAGATACATAAAAAT<br>R:CCGCTCGAGCTATTTTGTTCACTAAATATTTT
PF3D7_0800200-His	F:AAACCATGGGAAAGAAAAAACCGAAATCA<br>R:GGGCTCGAGTATATTCCATACATCCGATAT

Table S2. The primer sequences of RT-qPCR

Gene name	Primers
PF3D7_1372300	F:ATTCAAGGAATTTGGCACAG<br>R: CTGTTCAGTTACGCTCTCGAA
PF3D7_1205100	F:AAGTAGCAGGTCATCGTGGTT<br>R:TTCGGCACATTCTTCCATAA

Table S3. Binding kinetics of the PF3D7_1372300-GST and GST-tag to ATS using the ForteBio system.

Variant	KD(M)[a]	Kon(1/Ms)[b]	Kdis(1/s)[c]	R^2
1372300-GST	1.19E-07	6.33E+03	7.56E-04	0.9962
GST-tag	<1.0E-12	1.00E+04	<1.0E-07	0.1219

[a]KD: The equilibrium dissociation constant, calculated as Kdis/Kon.

[b]Kon: The binding rate.

[c]Kdis: The dissociation rate.

Table S4 Molecular dynamics datas of the Positions 1 to Position 5

Variant	Interaction Energy (kJ/mol)	RMSD (Å)	Number of H-Band	Number of Salt-Bridge
Position 1	-1122.133038	12.1089193	5.410494852	2.081368316
Position 2	-1230.713395	10.2997592	7.521641791	1.802238806
Position 3	-1965.027782	9.409817975	11.95051755	5.205251199
Position 4	-1157.593719	13.78770951	8.363332591	1.959010916
Position 5	-670.3124425	29.76553656	3.861900894	1.012591389

Table S5. Binding kinetics of the PF3D7_1372300-(27-129)-GST and PF3D7_1372300-(130-206)-GST to ATS using the ForteBio system.

Variant	KD(M)[a]	Kon(1/Ms)[b]	Kdis(1/s)[c]	R^2
PF3D7_1372300-(27-129)-GST	1.27E-07	6.63E+03	8.44E-04	0.9906
PF3D7_1372300-(130-206)-GST	2.26E-08	6.96E+03	1.58E-04	0.9726

[a]KD: The equilibrium dissociation constant, calculated as Kdis/Kon.

[b]Kon: The binding rate.

[c]Kdis: The dissociation rate.

[c]Kdis: The dissociation rate.
